# Histopathological, ultrastructural, and immunohistochemical examination of changes in the placenta as a result of severe preeclampsia

**DOI:** 10.1590/acb382023

**Published:** 2023-05-15

**Authors:** Çağdaş Özgökçe, Aydın Öcal, Işılay Seze Ermiş, Engin Deveci

**Affiliations:** 1 Zeynep Kâmil Hospital – Department of Obstetrics and Gynecology – Perinatology Department – Istanbul, Turkey.; 2 Harran University – Faculty of Medicine – Department of Obstetrics and Gynecology – Şanlıurfa, Turkey.; 3 Dicle University – Faculty of Medicine – Department of Histology and Embryology – Diyarbakır, Turkey.

**Keywords:** Pre-eclampsia, Histology, Immunohistochemistry, Placenta Diseases

## Abstract

**Purpose:**

To investigate the role of hypoxia-inducible transcription factor-1 alpha (HIF-1α) and angiogenetic factor endothelin-1 (ET-1) expression in regulating hypoxia and placental development by routine histopathological methods.

**Methods:**

Twenty preeclamptic and normal placentas were used. Placenta tissue pieces were examined histopathologically after routine paraffin follow-ups. HIF-1α and ET-1 proteins were examined immunohistochemically, and placental tissues were examined ultrastructurally.

**Results:**

Increase in syncytial proliferation, endothelial damage in vessels, and increase in collagen were observed in preeclamptic placentas. As a result of preeclampsia, an increase was observed in HIF-1α and ET-1 protein levels in the placenta. Dilatation of endoplasmic reticulum and loss of cristae in mitochondria were observed in trophoblast cells in preeclamptic placental sections.

**Conclusions:**

High regulation of oxygen resulting from preeclampsia has been shown to be a critical determinant of placentagenesis and plays an important role in placental differentiation, changes in maternal and fetal blood circulation, trophoblastic invasion, and syncytial node increase. It has been thought that preeclampsia affects secretion by disrupting the endoplasmic reticulum structure and induces mitochondrial damage, and that ET-1 may potentially help in the induction of stress pathways as a result of hypoxia in preeclampsia.

## Introduction

Preeclampsia used to be called the disease of theories, but clinical and experimental studies over the past decade have produced exciting breakthroughs that can improve diagnosis and even prediction, and lead to prevention and/or specific treatments. Some speculation continues on different theories such as changes in the morphology of the placenta, systemic inflammatory response, various hormones, and other proteins in the maternal cardiovascular system, changes in immune factors, cardiovascular inadaptations in pregnancy, and underlying maternal risks.

**Figure 1:**
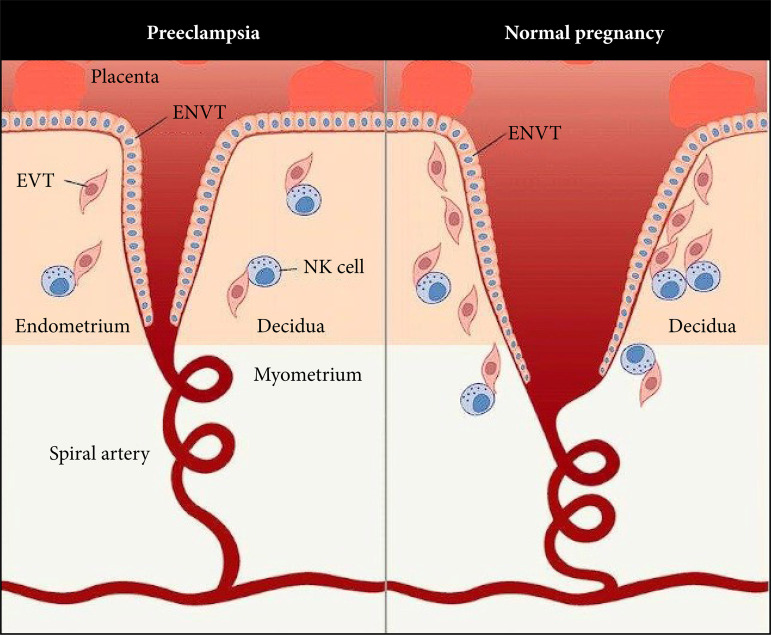
Schematic view of anatomical structures in both a normal pregnancy and preeclampsia.

EVT: extravillous trophoblast; ENVT: endovascular cytotrophoblast; NK cell: natural killer cell.

The most plausible theories about the cause of preeclampsia focus on the placenta and describe the disorder in two stages. The first stage happens when the placenta first develops. The placenta produces factors that enter the maternal circulation and are believed to be responsible for establishing the second stage. These problems occur in the second half of pregnancy and as the growing baby and placenta demand more resources from the mother’s body, resulting in visible maternal disease (high blood pressure, kidney, liver, brain, and clotting abnormalities). The anatomy of these changes is shown schematically in both a normal pregnancy and a patient with preeclampsia (Fig. 1). Overt disease is dependent not only on the influence of factors circulating through the placenta, but also on the mother’s health, including diseases that may affect the vascular system.

The placenta is an important tissue to understand the relationship between hypoxia (low O_2_ tension), organogenesis (organ development), and angiogenesis (blood vessel development). Preeclampsia results in significant adverse events such as maternal renal failure, liver involvement, organ dysfunction, uteroplacental insufficiency, and fetal growth retardation. Insufficiency of trophoblastic invasion of the placenta is important in the metabolism of preeclampsia. Changes in spiral arteries are disrupted in preeclampsia, cause hypoxia of the placenta, and decrease fetal blood circulation^1^.

It has been reported that abnormal development of placental villi, decreased proliferation of villous and extravillous cytotrophoblasts, and insufficient placental implantation cause trophoblast invasion^4^. Trophoblasts produce vascular endothelial growth factor and other angiogenic factors that support the placental vasculature with paracrine and autocrine action^5^. Decreased vascular endothelial growth fator (VEGF) and increased soluble fms-like tyrosine-containing kinase-1 (sFLT-1) production suggest a negative effect on placental vascular development^6^^,^ 7.

The most important member of the endothelin (ET) family is ET-1. It is synthesized by a number of cells, including endothelial cells and syncytiotrophoblasts of the placenta. ET secretion occurs structurally and by activation from depots in the Weibel-Palade bodies of endothelial cells^8^. Various stimuli such as angiotensin II, norepinephrine, thrombin, cytokines, growth factors, hypoxia, insulin, and free radicals have been reported to induce endothelial ET-1 release^9^^,^ 10.

The aim of this study was to investigate the role of HIF-1 and angiogenetic factor ET-1 expression in regulating hypoxia and placental development by immunohistochemical and ultrastructural methods in placental development and pathologies.

## Methods

### Patients and follow-up periods

Ethical approval of the study was obtained from Private Van Akdamar Hospital the Non-Interventional Clinical Trials Ethics Committee. The non-interventional clinical trials were obtained from the ethics committee. Among the participants of the study, there were 35-38-year-old pregnant women who were hospitalized in the Dicle University obstetrics clinic and whose consent was obtained. Postpartum placentas were removed at the week of birth.

In this study, 20 preeclamptic and 20 normal placentas (normotensive group) were used. Biochemistry results and clinical data were analyzed by taking blood samples from the patients. Preeclamptic patients with systolic > 140 mm/Hg and diastolic > 90 mm/Hg, and preeclamptic patients with proteinuria of 300 mg/24 hours were included in the determination of the preeclamptic group. The mean age of the patient group included in the study was 32, and the mean age of the control group was 28.

### Tissue processing and histological stainings

Tissue samples were taken from the maternal side of the placenta and fixed in 10% formaldehyde. Samples were dehydrated through ascending alcohol series and soaked in xylene and incubated in paraffin wax at 58°C and embeddedin in paraffin blocks. Five-μm thick sections were taken from paraffin-blocks of placental tissues. Sections were depraffinized in xylene and brought to distilled water. Sections were stained with hematoxylin and eosin staining and Trichrom-Masson staining^11^.

### Immunohistochemical technique

Paraffin sections taken on slides were kept in citrate solution for 3 × 5 minutes in a microwave oven for antigen retrieval. After cooling at room temperature for 10 minutes, the sections were washed with phosphate buffer solution. Then, they were treated with 3% hydrogen peroxide (H_2_O_2_) for 10 minutes. Sections rinsed in distilled water were washed with PBS (pH 7.6), and the sections were incubated with mouse monoclonal HIF-1α antibodies (1:100) and mouse monoclonal ET-1 antibodies (1:100). Sections were kept in secondary antibody solution (Biotinylated Goat Anti-Mouse, LabVision) for 10 minutes after been washed three times with PBS. Sections washed three times in PBS were treated with streptavidin peroxidase solution (Streptavidin Peroxidase, LabVision) for 3 × 5 minutes. After washing with PBS, 3-amino 9 ethyl carbazole chromogen solution was applied to the sections for 8 minutes. After washing the sections with distilled water, Mayer’s hematoxylin was applied for 2 minutes and counterstained and evaluated under a light microscope (Nikon)^12^^-^ 14.

### Electron microscopic technique

The dissection of the tissues was carried out in a 2.5% glutaraldehyde (pH = 7.2) solution with pH = 7.4 0.1 M Phosphate buffer. Initial fixation of the tissues was performed for 24 hours in 2.5% glutaraldehyde. Then, it was washed with pH = 7.4 0.1M phosphate buffer by changing it three times every 15 minutes. The second fixation was done in osmium tetroxide by rotating the rotator for 2 hours at room temperature. The tissue was washed three times and 15 times in phosphate buffer. In the dehydration process, 50, 70, 90% ethyl alcohol at +4°C two times for 15 minutes; 96 and 100% ethyl alcohol were also applied twice for 30 minutes.

Afterwards, the tissues were kept in propylene oxide twice for 30 minutes and a 1:1 mixture of propylene oxide and araldite in the rotator for 2 hours. After the tissues were kept in pure araldite in the rotator for one night, embedding was done. The polymerization process was carried out in an oven at 60°C for two days. Thin sections of 70-100 nm were taken on 200 mesh copper grids and stained in 2% uranyl acetate for 1 hour. After washing the grids with phosphate buffer, they were treated with lead acetate for 15 minutes. After treatment with lead acetate, it was washed again so that the samples were ready for examination under the electron microscope. Sections were examined with Jeol Brand JEM-1010 model transmission electron microscope in Dicle University Science and Technology Application and Research Center ,and cytopathological changes were photographed with GATAN brand, 782 side entry ES500W Erlangshen Model CCD camera connected to the same electron microscope.

### Statistical analysis

Statistical analysis was performed with IBM Statistics (version 25) software. The distribution of the data was examined. The independent sample t test was used for data with normal distribution, and the Mann-Whitney U test for non-distributed normally data. The value of *p* < 0.005 was considered significant.

## Result

**Figure 2:**
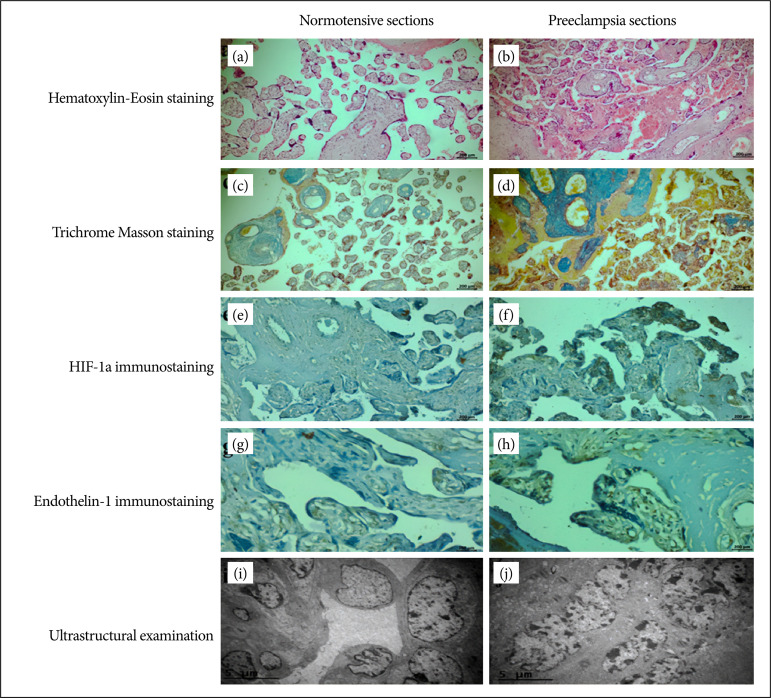
Presentation of routine histopathological methods in normotensive and preeclampsia groups.

Normotensive and preeclamptic patients’ data were compared. Data of preeclamptic patients differed compared to the control group in terms of gestational week, birth weight, and urinary protein parameters, and this difference was statistically significant (Table 1 and Fig. 2).

**Table 1:** Comparison of patient characteristics and biochemical parameters between normotensive and preeclampsia groups*.

Parameters	Normotensive group (mean ± SD)	Preeclampsia group (mean ± SD)	p-value
Maternal age	24.95 ± 4.80	25.35 ± 4.98	0.797
Gravida	2.75 ± 1.07	2.70 ± 1.22	0.891
Parity	1.55 ± 1.00	1.20 ± 0.95	0.264
Pregnancy weight gain	9.75 ± 2.27	10.00 ± 1.81	0.702
Maternal BMI	23.35 ± 2.35	24.25 ± 2.59	0.257
Dad height	175.50 ± 5.09	176.00 ± 4.87	0.753
Birth week	39.25 ± 0.85	34.60 ± 1.79	0.001*
Birth height	49.65 ± 0.99	47.65 ± 1.04	0.995
Birth weight	3,315.90 ± 288.20	2,206.00 ± 166.11	0.001*
Hemoglobin	10.65 ± 1.04	11.65 ± 1.04	0.995
Hematocrit	32.10 ± 3.31	34.95 ± 3.38	0.781
Platelet	244.50 ± 67.51	179.00 ± 71.35	0.904
Uric acid	3.62 ± 0.98	5.66 ± 1.07	0.975
Creatinine	0.41 ± 0.14	0.65 ± 0.17	0.314
Subordinate	14.20 ± 3.41	48.35 ± 16.72	0.001*
Lower	14.65 ± 2.80	57.40 ± 18.55	0.001*
Urine protein	161.25 ± 52.46	871.25 ± 870.87	0.001*
Albumin	3.33 ± 0.32	2.84 ± 0.21	0.307

SD: standard deviation; BMI: body mass index;

* p < 0.005 was considered significant by the independent sample t test.

### Histopathologic examination

Hematoxylin and eosin staining, Trichrome Masson staining, HIF-1α and ET-1 immunostainings and electron microscopy examination are shown in Fig. 2.

HIF-1α: hypoxia-inducible transcription factor-1 alpha.

### Hematoxylin and eosin staining findings

In normotensive group, apart from the chorionic villi, the blood vessel endothelial cells, in which syncytial cells have a prismatic appearance, flattened and connective tissue cells were solitarily dispersed, while there was no bleeding in the intervillous area (Fig. 2a)–hematoxylin-eosin staining, bar: 200 μm, magnification: 20X.

In preeclampsia group, an increase in chorionic syncytial nodes was observed in the transversal section of chorionic villi. A long xxxx with the dilatation of the blood vessels inside the chorionic villi, free scattered erythrocytes and hyperplasia were observed in the endothelial cells. In areas with some chorionic villi, an increase in fibrinoid tissue between the two villi, an increase in connective tissue in the villi and an increase in leukocytes in the intervillous area were detected (Fig. 2b)–hematoxylin-eosin staining, bar: 200 μm, magnification: 20X.

### Trichrome Masson staining findings

In normotensive group, it was observed that the mucous connective tissue structure around the blood vessels of the stem villi was regular, and there was no change in the villi (Fig. 2c)–Trichrome Masson staining, bar: 200 μm, magnification: 20X.

In preeclampsia group, a significant increase in syncytial nodes, a significant thickening of the blood vessel basement membrane structure, and an increase in the collagenized areas were observed with dense fibroid tissue (Fig. 2d)–Trichrome Masson staining, bar: 200 μm, magnification: 20X.

### HIF-1α immunostaining findings

In normotensive group, negative HIF-1α expression was observed in the syncytial cells on the outer side of the stem villi and chorionic villi and in blood vessel endothelial cells, while HIF-1α showed a positive reaction in hofbauer and some connective tissue cells with macrophage characteristics (Fig. 2e)–HIF-1α immunostaining, bar: 200 μm, magnification: 20X.

In preeclampsia group, HIF expression was observed as clusters in the fibroid structures inside the chorionic villi and positive HIF expression in the syncytial nodes (Fig. 2f)–HIF-1α immunostaining, bar: 200 μm, magnification: 20X.

### Endothelin-1 immunostaining findings

In normotensive group, ET-1 expression was weakly expressed in endothelial cells in the blood vessel wall and some macrophage cells (Fig. 2g)–ET-1 immunostaining, bar: 200 μm, magnification: 20X.

In preeclampsia group, ET-1 expression was increased in cytotrophoblast cells, endothelial cells and Hoffbauer cells (Fig. 2h). ET-1 immunostaining, bar: 200 μm, magnification: 20X.

### Ultrastructural examination findings

In normotensive group, in the transversal section of the chorionic villi, cytotrophoblast cell nuclei in heterochromatin structure and mitochondria and endoplasmic reticulum structures in the cytoplasm appeared normal. The nuclei of the blood vessel endothelial cells were protruding towards the heterochromatin and lumen, and the basement membrane thicknesses were normal (Fig. 2i)–uranyl acetate staining, bar: 5 μm, magnification: 20X.

In preeclampsia group, pycnosis and coarse-grained chromatin structures were observed in the cytotrophoblast cell nuclei. Dilatation in the endoplasmic reticulum structures in the cytoplasm, degenerative changes in the mitochondria and loss of cristae were observed (Fig. 2j)–uranyl acetate staining, bar: 5 μm, magnification: 20X.

## Discussion

Preeclampsia is a hypertensive disease that presents marked hypertension and proteinuria, complicating pregnancy. Placental syncytium is the most important key in the pathogenesis of preeclampsia. Preeclampsia is one of the most feared complications of pregnancy and usually occurs as new-onset hypertension and proteinuria in the third trimester. It causes fatal consequences for the mother and the fetus. In the early stage of preeclampsia, mild hypoxia or oxidative stress can cause activation of trophoblast proliferation to cope with the transient syncytial deficiency. Hypoxia is one of the important causes of preeclampsia, a pregnancy-specific syndrome characterized by maternal hypertension and proteinuria. Transformation of maternal spiral arteries occurs during placental development in the first weeks of pregnancy, and this transformation is disrupted in preeclampsia, causing a decrease in placental perfusion that triggers the inflammatory response. Starting from the second trimester of pregnancy, the inflammatory response develops. As a result of damage to the endothelial region of the vessels in the maternal circulation, there are systemic vasoconstriction, and hypertension increase (Table 1, Fig. 2)^15^^-^ 18.

ET-1 is not only an independent predictor of both blood pressure increase and proteinuria in preeclampsia, but also a renin suppressor^19^. The increase in ET-1 is the direct result of VEGF inactivation or inhibition. It was emphasized that since ET-1 itself triggers oxidative stress in the placenta, this may lead to increased production of placental factors such as soluble fms-like tyrosine kinase-1 (sFLT-1)^20^. The ET system, in which preeclampsia is a complex multifactorial disease that potentially requires multiple levels of therapeutic intervention, has been reported to be a pathway that may be the cause of hypertension, renal toxicity, and RAS suppression in preeclampsia.

It has been shown that high maternal ET-1 production causes preeclampsia-like phenotypes during pregnancy and affects both the initial stage of trophoblast differentiation/invasion and the maternal peripheral vasculature during late pregnancy^21^. It has been shown to induce hypertension experimentally in preeclampsia through the production of the anti-angiogenic protein sFLT-1, inflammatory cytokines, and the agonistic angiotensin II type-1 receptor autoantibodies ET-1^22^^,^ 23. These factors cause endothelial cell damage. In our study, hyperplasia in endothelial cells as a result of preeclampsia and increase in ET-1 expression as a result of endothelial damage caused the induction of hypertension (Fig. 2).

In a study with patients with preeclampsia complicated by ultrastructural examination, focal syncytial necrosis, rough endoplasmic reticulum enlargement, decreased pinocytotic activity and decreased number of secretory droplets were demonstrated. In areas in which syncytial necrosis was detected, the cells were swollen, and mitochondria structure was shown. An increasing degree of basal interdigitation has been demonstrated in the plasma membrane, noted especially in placentas with severe preeclampsia^24^. An increase in basal lamina thickness and intervillous collagen and a decrease in the volume and complexity of mitochondria have been noticed, with extensive damage to the syncytium in hypoxic conditions. They emphasized that hypoxia conditions induce a marked thinning and vacuolation in syncytiotrophoblasts, as well as inducing increased folding of syncytial basement membranes, clubbing of microvilli and aggregation of nuclear chromatin. As a result, it has been reported that it also supports cellular degeneration, which causes the loss of organelles required for syncytial function^25^.

In our study, pycnosis and large chromatin structures were observed in cytotrophoblast cells, as well as dilatation in the endoplasmic reticulum in the cytoplasm, loss of cristae in mitochondrial structures and syncytial function (Figure 2).

## Limitations

Patient number could be increased. Western blot could be performed to quantitatively support immunohistochemical staining.

## Conclusion

High regulation of HIF-1α oxygen resulting from preeclampsia has been shown to be a critical determinant of placentagenesis and plays an important role in placental differentiation, changes in maternal and fetal blood circulation, trophoblastic invasion, and syncytial node increase. It has been thought that preeclampsia affects secretion by disrupting the endoplasmic reticulum structure and induces mitochondrial damage, and that ET-1 may potentially help in the induction of stress pathways as a result of hypoxia in preeclampsia.

## Data Availability

All generated data were presented in this study.
